# Intralymphatic immunotherapy of pollen-induced rhinoconjunctivitis: a double-blind placebo-controlled trial

**DOI:** 10.1186/s12931-016-0324-9

**Published:** 2016-01-27

**Authors:** Terese Hylander, Olivia Larsson, Ulla Petersson-Westin, Mia Eriksson, Susanna Kumlien Georén, Ola Winqvist, Lars-Olaf Cardell

**Affiliations:** Division of ENT Diseases, Department of Clinical Sciences, Intervention and Technology, Karolinska Institutet, Stockholm, Sweden; Laboratoy of Clinical Experimental Allergy Research, Department of Otorhinolaryngology Malmö, Lund University, Skåne University Hospital, Malmö, Sweden; Department of ENT Diseases, Karolinska University Hospital, Stockholm, Sweden; Department of Medicine Solna, Translational Immunology Unit, Karolinska University Hospital Solna, Stockholm, Sweden

**Keywords:** Allergic rhinitis, Allergen-specific immunotherapy, Intralymphatic immunotherapy, Seasonal allergisc rhinitis, IgG4

## Abstract

**Background:**

Allergen-specific immunotherapy represents the only disease-modifying treatment for allergic diseases. We and others have previously demonstrated that intralymphatic immunotherapy (ILIT), a less time-consuming alternative to conventional subcutaneous immunotherapy (SCIT), is safe and effective. However, this has recently been disputed. The aim of this study was therefore to expand our previous trial, further assessing the safety and efficacy of ILIT.

**Methods:**

Thirty-six patients with pollen-induced rhinoconjunctivitis were randomised to receive three intralymphatic inguinal injections of active allergen (1000 SQ-U birch- or grass-pollen) or placebo. Clinical effects, safety and circulating immunological markers were assessed before, 4 weeks after treatment and at the end of the consecutive pollen season.

**Results:**

No moderate or severe reactions were recorded following ILIT. Patients receiving active ILIT experienced a significant improvement in self-recorded seasonal allergic symptoms, as compared to placebo (*p* = 0.05). In a subgroup of these patients (“improved”), a reduction in nasal symptoms following nasal allergen provocation was also demonstrated. No changes in total IgE or IgG_4_ were found. However, the affinity of allergen specific IgG_4_ following active treatment was significantly increased, as compared to non-improved patients (*p* = 0.04). This could be correlated with clinical improvement, on an individual level.

**Conclusions:**

This double-blinded placebo-controlled study confirms that ILIT is a safe and effective treatment for pollen-induced rhinoconjunctivitis, markedly reducing seasonal allergic symptoms.

**Trial registration:**

EudraCT: 2009-016815-39

**Electronic supplementary material:**

The online version of this article (doi:10.1186/s12931-016-0324-9) contains supplementary material, which is available to authorized users.

## Background

Allergic rhinitis is a growing public health problem, affecting over 400 million people worldwide [1]. Currently, allergen-specific immunotherapy (AIT) represents the only disease-modifying treatment, diminishing symptoms, improving quality of life, preventing new sensitisations and reducing the risk of asthma development [2, 3]. The golden standard treatment is subcutaneous immunotherapy (SCIT), which shows long-term benefit for the treatment of allergic rhinitis, conjunctivitis and asthma [3, 4]. Despite this, only 5 % of patients undergo this therapy, due to frequent injections, risk of adverse effects and the long duration of treatment [2]. A more recent, non-invasive route of administration is sublingual immunotherapy (SLIT). Though efficacious, SLIT is associated with reduced compliance due to the long period of self-medication [5].

Intralymphatic immunotherapy (ILIT) is an emerging form of AIT that involves three injections of allergen over a period of 12 weeks. This form of AIT directs lower doses of allergen to the highly immunocompetent lymph node, in an effort to maximise chances for tolerance induction, while minimising the risk for adverse effects. In the pioneering study in 2008, ILIT was shown to induce long-lasting allergen tolerance, equivalent to that of SCIT, but with fewer adverse events [6]. Since then, ILIT has demonstrated clinical efficacy against allergy to cat dander and, recently, against grass-pollen induced rhinoconjunctivitis in adolescents and young adults [7, 8].

In a recent small double-blind placebo controlled study, we too demonstrated that ILIT against grass pollen resulted in a significant improvement of patient-recorded symptoms during the pollen season, while injection-associated discomfort levels were comparable to that of SCIT [9]. However, the clinical efficacy of ILIT is currently disputed [10]. Consequently, the aim of the present study was to expand our first trial to determine if the safety and efficacy of ILIT persisted in a larger cohort of patients.

## Methods

### Study population and eligibility criteria

Study subjects were recruited amongst patients at the allergy department of Skåne University Hospital, Malmö, Sweden. Eligible patients were aged between 18 and 65 and had moderate to severe allergic birch/grass pollen-induced rhinoconjunctivitis, with symptoms including itchy nose and eyes, sneezing, nasal congestion and secretion. Allergy was verified by positive skin prick tests (SPTs), presence of serum-specific IgE antibodies towards birch and/or grass (minimum 0.35 kU/L) and positive nasal provocation tests (NPTs). Sample size was based on our previous study [9]. All eligible participants recruited during the recruitment period were enrolled in the study.

General contraindications were pregnancy or nursing, planning for pregnancy, autoimmune and collagen disease, cardiovascular disease, current persistent asthma (not intermittent asthma), upper airway disease (non-allergic sinusitis, nasal polyps), chronic obstructive and restrictive lung disease, hepatic and renal disease, cancer, previous immune- or chemotherapy, major metabolic disease, alcohol or drug abuse, mental incapability (to cope with the study) or medication with a possible side-effect of interfering with the immune response.

### Study design

This study was a parallel double-blind placebo-controlled trial, performed at the allergy department of Skåne University Hospital, Malmö, Sweden.

In total 36 patients were enrolled. Fifteen patients were recruited in the first cohort (September 2010 to September 2011) and have been previously defined [9]. Twenty-one patients were recruited in the second cohort (September 2011 to September 2012).

At the first visit (visit 1, out of pollen season, 2010 or 2011), patient eligibility was determined, SPTs and NPTs were performed and blood was sampled (further details in Additional file 1). After approximately one week, included patients were randomly allocated to receive either placebo (*n* = 15) or active (*n* = 21) intralymphatic treatment. At visits 2–4 (September 2010 – January 2011 or September 2011 – January 2012), the study subjects received three 0.1 ml injections with either placebo (Alutard, ALK Abéllo, Horsholm, Denmark) or 1000 SQ-U of standardised, aluminium hydroxide adsorbed, depot birch- or grass-pollen vaccine (Alutard, ALK Abéllo) at 3- to 4-week intervals. Based on the outcome of the allergy tests, patients were challenged and vaccinated with either birch or grass (mono-sensitised). Patients returned approximately 4 weeks after the last injection (visit 5, February 2011 or February 2012) and after the consecutive pollen season (visit 6, September 2011 or September 2012), and were evaluated as per visit 1. At visit 6, patients were additionally asked to complete a questionnaire regarding their seasonal allergic symptoms as compared to the previous pollen season. One patient did not complete the treatment, due to a non-severe adverse event (local urticaria). Emergency envelopes remained unbroken.

### Randomisation, allocation and blinding

Participants were randomly assigned to one of two treatment groups following a simple randomisation procedure with opaque, sealed envelopes. The randomisation procedure was generated by independent biomedical assistants. The vaccines used were pre-packed, blinded and allocated according to the randomisation sequence by independent staff with no connection to the study, and thus both patients and physicians were blind. Recruitment was performed by UPW. All those involved in the study, including participants, care-givers and those assessing outcomes, were blinded after assignment to interventions.

### Ethics, consent and permissions

The study was approved by the local ethics committee (ref. ID 2009/714) and all participants gave their written informed consent.

### Intralymphatic injections

A superficial inguinal lymph node in either the left or right groin was aseptically injected using a 25-gauge needle and ultrasound guidance. The superficial lymph nodes in the groin were identified as hypoechoic nodules with a diameter of 0.5 to 1.5 cm. The same side and, as far as possible the same node, was targeted during all three injections. Aspirations were made before the injections to avoid inadvertent intravascular administration. The peak expiratory flow (PEF) was measured before and after each injection, and all patients were monitored at the ward for no less than 60 min after each injection. The trial staff recorded all signs of local and/or systemic reactions in conjunction with the injections. The patients were subsequently asked to record and report all indications of late reactions for the following 24 h.

### Trial outcomes

#### Primary outcome measures

The primary outcome measure for the study was the change in pollen season-associated allergic symptoms. At the end of the first allergy season, after treatment had been given (visit 6), patients were asked to compare their most recent seasonal allergic symptoms with the symptoms they experienced during the pollen season prior to treatment. To this end, a visual analogue scale (VAS), ranging from 0 (unchanged symptoms, no improvement) to ten (total symptom relief, complete recovery), was used, as previously described [11].

#### Secondary outcome measures

The secondary outcome measures of the study were 1) the safety of intralymphatic injections; 2) the change in nasal symptom score (NSS) following NPT (comparisons of NSS after NPT before treatment, after treatment and after the consecutive pollen season), as previously described [9]; 3) the change in circulating immunoglobulin (IgE and IgG_4_) levels (comparisons of levels before treatment, after treatment and after the consecutive pollen season); and 4) change in circulating inflammatory cells (comparisons of levels before treatment, after treatment and after the consecutive pollen season).

#### Additional secondary outcomes

Blood samples acquired at visit 6 (after the pollen season following treatment) were re-analysed following unblinding of the study, to determine alterations in IgG_4_ affinity. This was due to the appearance of two distinct sub-populations in the active ILIT group (“improved” and “non-improved”; see Results), which demonstrated no difference in IgG_4_ levels. Further analysis was consequently performed, with the aim to immunologically discriminate between these groups.

#### Tertiary outcomes

As a tertiary outcome, patients were asked to report their usage of allergy medication during the pollen season immediately after ILIT, with their use in the allergy season prior to the start of treatment. Patients were asked to report a reduction in use, an increase in use or a no change in use, in terms of their use of anti-histamines, corticosteroid nasal spray and eye drops.

### Methods used for assessment of secondary and additional trial outcomes

Methods used for the assessment of trial outcomes, including nasal provocation test, flow cytometry, and assessment of IgG_4_ affinity can be found in the supplementary material (Additional file 1).

## Statistical analysis

Statistical analysis was performed using GraphPad Prism 5.01 software (San Diego, CA, USA). Distribution of data was assessed using the D’Agostino and Pearson omnibus normality test. Parametric data was analysed using a Student’s unpaired *t*-test (unpaired observations) or repeated measures ANOVA followed by Dunnet’s multiple comparison post-test (paired observations). Non-parametric paired observations were analysed using the Friedman test, followed by Dunn’s multiple comparison post-test. IgG_4_ affinity was analysed by two-way ANOVA, followed by the uncorrected Fisher’s LSD test. Correlation was determined by calculating the Pearson’s correlation coefficient. Data are represented as mean ± SEM. A *p*-value of 0.05 or less was considered statistically significant.

## Results

### Patients

**Fig. 1:**
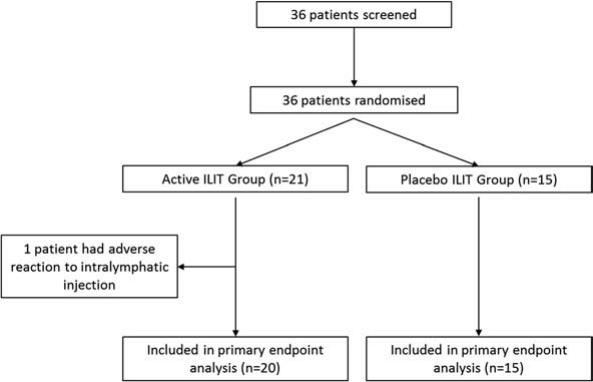
Flow diagram of study cohort. ILIT: Intralymphatic immunotherapy

A flow-diagram showing enrolment/screening, allocation, and patient follow-up is depicted in Fig. 1. Thirty-six participants were screened between September 2010 and September 2011. They all demonstrated positive SPT, NPT and serum IgE and were therefore enrolled in the trial. Fifteen patients were enrolled in September 2010 [9]; 21 patients were enrolled in September 2011. Twenty-one patients received active ILIT (seven in the 2010 study, 14 in the 2011 study) and 15 patients received placebo (eight in the 2010 study, seven in the 2011 study). One patient receiving active ILIT demonstrated localised urticaria following the first intralymphatic injection and did not wish to further participate in the study. The demographics of participants are depicted in Table 1.

**Table 1:** Baseline characteristics of patients included in the study

	Active ILIT	Placebo ILIT
Number of participants	21	15
Age (y), median (range)	30 (20–52)	30 (20–54)
Gender (male vs. female)	13:8	9:6
Allergen-specific IgE (kU/L)	4.4 – 100.0	0.47 – 78.6
Birch vs. grass vaccination	8:13	6:8

### Primary End-Point

**Fig. 2:**
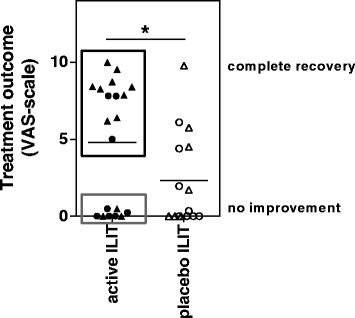
Patient-recorded treatment outcome following three intralymphatic injections with active ILIT or placebo. Patients compared seasonal allergic symptoms after treatment, with symptoms prior to treatment. A VAS-score of 0 indicates no improvement, whereas a score of 10 indicates complete recovery. The black box signifies “improved” patients with a VAS-score above 5; the grey box signifies “non-improved” patients with a VAS-score below 1. Circles represent patients with an allergy towards birch pollen; triangles represent patients with an allergy towards grass pollen. *p < 0.05 using an Unpaired *t* test. ILIT: Intralymphatic immunotherapy

At the end of the pollen season following treatment, subjects were asked to compare their seasonal allergic symptoms with the allergic season prior to the start of treatment. A clear improvement in relation to baseline was observed in the active ILIT group (VAS-score: 4.78 ± 0.9) (Fig. 2). This improvement was significantly more marked than the effects recorded in the placebo group (*p* = 0.047). However, no substantial reduction in medication could be verified in the active ILIT group (Additional file 2).

Evaluation of treatment outcome additionally resulted in the emergence of two clear sub-populations among the patients who received active ILIT: one group of 12 patients that reported a clear improvement in symptoms (VAS-score > 5) and a group of 8 patients that reported no improvement (VAS-score < 1) (Fig. 2). No patients reported a VAS-score between 1 and 5.

### Secondary End-Point

#### Safety assessment of intralymphatic allergen administration

The number of local drug-related reactions (e.g. lymph node swelling, itch and redness close to the proximity of the injection site) was higher in the active group than in the placebo group. One patient recorded mild side-effects at the first injection (localised redness and urticaria), which resulted in the individual’s withdrawal from the study. However, no moderate or severe reactions were recorded (Table 2). At the follow-up visit, approximately 4 weeks after the final injection, all drug-related reactions were resolved.

**Table 2:** Adverse events associated with intralymphatic injections

	Active ILIT (61 injections)		Placebo ILIT (45 injections)	
	Early Reactions	Late Reactions	Early Reactions	Late Reactions
Local Lymph Node Swelling	Mild: 6	Mild: 14	Mild: 1	Mild: 1
	Moderate: 0	Moderate: 0	Moderate: 0	Moderate: 0
	Severe: 0	Severe: 0	Severe: 0	Severe: 0
Local Itch	Mild: 6	Mild: 11	Mild: 2	Mild: 1
	Moderate: 0	Moderate: 0	Moderate: 0	Moderate: 0
	Severe: 0	Severe: 0	Severe: 0	Severe: 0
Local Redness	Mild: 11	Mild: 21	Mild: 0	Mild: 0
	Moderate: 0	Moderate: 0	Moderate: 0	Moderate: 0
	Severe: 0	Severe: 0	Severe: 0	Severe: 0
Nasal Symptoms	Mild: 1	Mild: 1	Mild: 4	Mild: 4
	Moderate: 0	Moderate: 0	Moderate: 0	Moderate:0
	Severe: 0	Severe: 0	Severe: 0	Severe: 0
Pulmonary Symptoms	Mild: 0	Mild: 0	Mild: 0	Mild: 0
	Moderate: 0	Moderate: 0	Moderate: 0	Moderate: 0
	Severe: 0	Severe: 0	Severe: 0	Severe: 0
Urticaria and angiooedema	Mild: 1	Mild: 4	Mild: 0	Mild: 1
	Moderate: 0	Moderate: 0	Moderate: 0	Moderate: 0
	Severe: 0	Severe: 0	Severe: 0	Severe: 0
Abdominal Symptoms	Mild: 0	Mild: 1	Mild: 0	Mild: 1
	Moderate: 0	Moderate: 0	Moderate: 0	Moderate: 0
	Severe: 0	Severe: 0	Severe: 0	Severe: 0

#### Circulating immunological markers

**Fig. 3:**
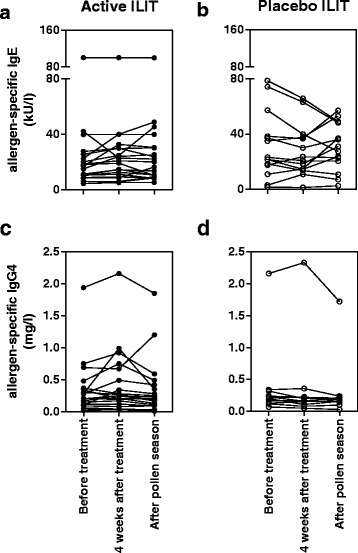
Levels of circulating immunoglobulins. IgE (**a**-**b**) and IgG_4_ (**c**-**d**) were measured before treatment, four weeks after treatment and after the consecutive pollen season in patients treated with active ILIT (**a**, **c**) or placebo (**b**, **d**). Analysis was performed using a repeated measures ANOVA. ILIT: Intralymphatic immunotherapy

No significant differences in total serum levels of IgE or IgG_4_ were evident in the active ILIT group or placebo group (Fig. 3). In addition there was no significant increase in CD4^+^CD25^+^FoxP3^+^ lymphocytes in the active ILIT or placebo group (data not shown).

#### Nasal symptom scores

**Fig. 4:**
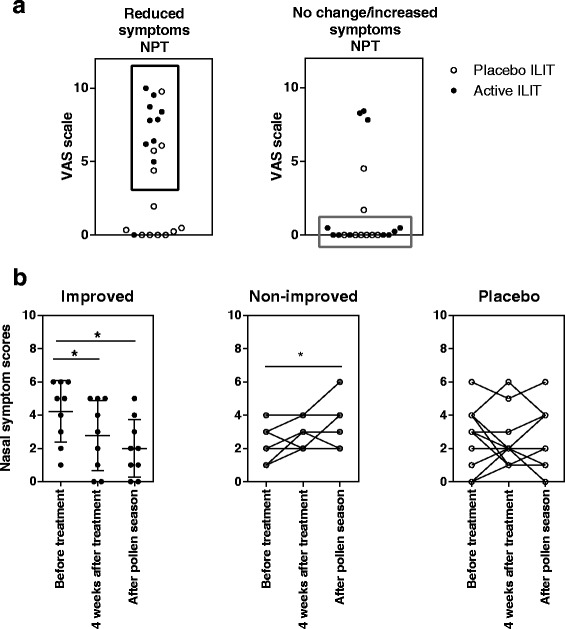
Identification and nasal symptom scores of improved and non-improved patients. **a** Identification of patients reporting change in allergic symptoms 30 min after nasal allergen provocation (NPT), as well as change of seasonal allergic symptoms (VAS-score 0 = no improvement; VAS-score: 10 = complete recovery) after treatment. “Improved” patients are depicted with a black box. Non-improved patients are depicted with a grey box. **b** Self-reported allergic symptoms 30 min after nasal allergen provocation before treatment, four weeks after treatment and following the consecutive pollen season in improved, non-improved and placebo patients *p < 0.05 using a repeated measures ANOVA followed by a Dunnet’s multiple comparison post-test. ILIT: Intralymphatic immunotherapy

No significant change in NSS following NPT could be seen in the active ILIT or placebo groups (data not shown). However, nine out of 12 patients in the active ILIT group with a VAS-score over five demonstrated a reduction of allergic symptoms upon NPT after treatment (“improved” sub-population). In contrast, seven out of eight patients in the active ILIT group with a VAS-score under 1 demonstrated no reduction of allergic symptoms upon NPT after treatment (“non-improved” sub-population) (Fig. 4a).

Further analysis revealed that improved patients demonstrated a reduction in NSS following NPT 4 weeks after treatment (*p* = 0.014) and after the consecutive pollen season (*p* = 0.015) (Fig. 4b). These reductions were not evident in the eight non-improved patients, or in the placebo group. In contrast, an increase in NSS following NPT was evident in non-improved patients, after the consecutive pollen season (*p* = 0.011)

#### “Improved” vs “Non-improved” IgG_4_ levels and IgG_4_ affinity

**Fig. 5:**
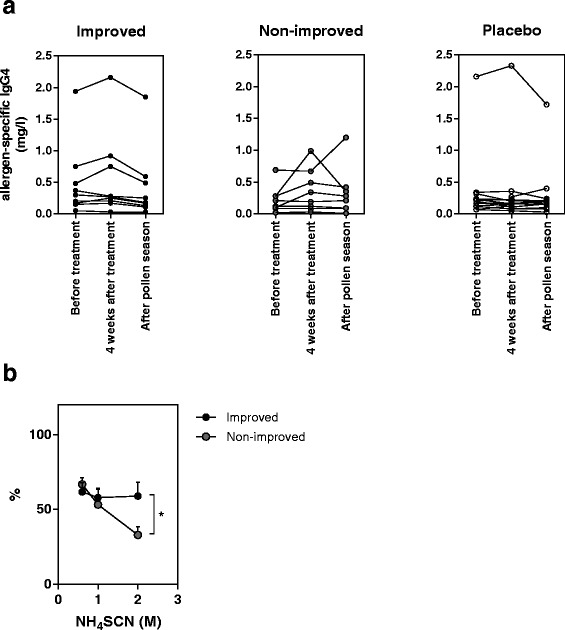
Change in IgG_4_ levels and affinity in improved and non-improved patients. **a** Levels of circulating IgG_4_ in improved and non-improved actively treated patients, as well as placebo patients, before treatment, four weeks after treatment and following the consecutive pollen season. Analysis was performed using a repeated measures ANOVA. **b** IgG_4_ affinity in improved and non-improved patients, measured and presented as percent allergen-bound IgG_4_ with increasing concentrations of ammonium thiocyanate (NH_4_SCN). *n* = 3–7 *p < 0.05 using two-way ANOVA followed by Fisher’s LSD

Analysis of IgG_4_ levels in the nine improved and 8 non-improved patients, as well as the placebo group, revealed no changes in levels of IgG_4_ before treatment, after treatment and after the consecutive pollen season (Fig. 5a).

In contrast, analysis of IgG_4_ affinity in samples taken after the consecutive pollen season demonstrated that allergen-specific IgG_4_ affinity was significantly increased in improved patients (*p* = 0.035), as compared to non-improved patients (Fig. 5b).

In addition, a significant and positive correlation between IgG_4_ and both VAS score (r^2^ = 0.42, *p* = 0.04) and reduction in nasal symptom score (r^2^ = 0.48, *p* = 0.03) was found.

### Tertiary End-Point

No substantial reduction in medication (anti-histamines, local nasal steroids) could be verified in the active ILIT group (Additional file 2).

However, improved patients reported a reduction in the use of all allergy medication in the allergy season following treatment. This was not evidenced in the non-improved, actively treated patients. Improved patients demonstrated a stronger reduction in the use of corticosteroid nasal spray and eye drops, but not anti-histamines, as compared to the placebo group (Additional file 2).

## Discussion

In a recent study, we demonstrated that ILIT is a safe and effective administration route for the induction of allergen tolerance and treatment of nasal allergic inflammation [9]. In the present study, we have added a new cohort of study subjects and together they reinforce the hypothesis that ILIT is effective in reducing allergic symptoms and that it is not associated with severe adverse events. In addition, we highlight a role for increased allergen-specific IgG_4_ affinity in successful ILIT.

During the past few years, ILIT has come forward as a less time-consuming and more cost-effective alternative to conventional subcutaneous allergen-specific immunotherapy (SCIT) for treatment of pollen-induced rhinoconjunctivitis. It has provided promising clinical results in combination with an excellent safety profile [6, 9], likely related to the drastic reduction in amount of administered allergen. This present study demonstrates that, as compared to placebo, active ILIT treatment results in improvement of seasonal allergic symptoms, without any moderate or severe adverse reactions. This study further supports ILIT as a safe and effective treatment strategy for patients with pollen-induced rhinoconjunctivitis. Indeed, the 60 % success rate is comparable to the approximate 70 % success rate for SCIT [12]. Although it is evident that five out of eight placebo patients responded to treatment, it is worth noting that the number needed to treat (NNT) value for ILIT, which takes into account responders and non-responders in both active and placebo groups, is 7.5. This NNT value is much lower than those calculated for more common treatments of allergic rhinitis, such as anti-histamines [13].

The somewhat lower success rate of ILIT as compared to SCIT may be related to the technical aspects of allergen injections. Unlike SCIT, ILIT uses complex medical equipment and involves higher technical precision, as targeted nodes are relatively small (0.5–1.5 cm). Despite the use of a fine needle and ultrasound guidance, it can on occasion be difficult to penetrate the capsule surrounding the lymph node, which may lead to inadequate administration of allergen.

Despite repeated promising results, the overall efficacy of ILIT against grass-pollen-induced allergic rhinitis has recently been questioned [10]. In a double-blind placebo-controlled trial, 15 patients receiving active ILIT against grass pollen showed immunological indications of tolerance, but no clinical improvement. As recently argued [14, 15], these results were likely due to the interval between injections. Witten et al [10] injected allergen every 2 weeks, which, unlike a 4-week interval, does not allow sufficient time for the development of non-interfering waves of antigen-specific immune responses [16]. The present study, where injections were given at 4-week intervals, again highlights the importance of correct time interval between injections.

In this study, two distinct sub-populations could be observed among the actively treated patients. Patients in the first sub-population reported a clear improvement of their allergic symptoms, as characterised by reduced nasal allergen reactivity, decreased usage of allergy medication (anti-histamines, local steroids, eye drops) and self-recorded improvement (VAS-scale). In contrast, patients in the second sub-population experienced no resolution of symptoms. The reason for the emergence of two sub-populations is unknown and may, at least in part, be related to technical difficulties during intralymphatic injection, resulting in extra-nodal deposits of allergen and an ineffective tolerance induction. Nevertheless, the sub-populations firstly demonstrate the usefulness of the nasal provocation test (NPT) as a read-out of clinical efficacy, as nine out of 12 patients with self-reported improvement in season allergic symptoms (VAS-scale) also reported a reduction in symptoms following NPT. Secondly, these populations give insights into the underlying immunological mechanisms associated with efficacious ILIT.

The present study, as well as the aforementioned study by Witten et al [10], both suggest that immunological alterations associated with tolerance induction, including elevated T-regulatory cells and cytokines, and IgG_4_ levels in blood, are not prerequisites for clinical efficacy of ILIT. Witten et al [10] noted increases in T-regulatory cells, IL-10 production and IgG_4_ following active ILIT, but demonstrated no clinical improvement when compared to placebo. In contrast, we demonstrated clinical improvement in the absence of increases in circulating T-regulatory cells and total serum IgG_4_. The reason for this is unclear, but may be related to the short time interval between vaccination and blood sampling. However, we demonstrated that clinical improvement was associated and significantly correlated with increased allergen-specific IgG_4_ affinity. Indeed, as previously suggested [14] affinity of B- and T-cell receptors is likely a very important parameter for the induction of tolerance to allergen and subsequent efficacious therapeutic responses.

There is an intricate interaction between B- and T-cells in the specialised lymph node compartment. B-cells bind the injected allergen via the cell-surface B-cell receptor (BCR), which leads to receptor mediated endocytosis, processing and peptide loading. Subsequently, allergen-derived peptides are presented via the MHC class II pocket to allergen-specific CD4^+^ T-helper cells. This B-cell induced activation of T-cells, reciprocally, allows for T-helper cell-licensed class switching and affinity maturation of IgG [17]. In the case of the present study, the injected composition resulted in increased affinity of IgG_4_, avoiding IgE production. The reason for IgG_4_ preferences in the present study is likely the use of adjuvant, as well as the compartment for the delivery of allergens, lymph nodes being more prone to IgG responses, as compared to airway responses to pollen allergens.

Furthermore, competition for antigen, which occurs more effectively when low concentrations of antigen is present in lymph follicles, is vital for the positive selection of high affinity B-cells, and this phenomenon has similarly been suggested for selection of high affinity T-cells [18]. This suggests that a 2-week-interval between injections, where concentrations of allergen in lymph nodes is consistently high, may induce clear B- and T-cell responses, as seen by increased serum levels of total IgG_4_ levels and allergen-induced intracellular T-cell cytokine production [10], but with a low affinity maturation, resulting in poor clinical efficacy. Indeed, previous studies have noted that functionality of IgG_4_, rather than levels, more closely relate to clinical efficacy in AIT [19, 20]

Although this study clearly indicated that ILIT does improve clinical symptoms of pollen-induced rhinoconjunctivitis, particularly in the improved sub-population, there are a number of limitations. Firstly, the use of a VAS scale for patient recorded outcome has been debated, in particular in long-term studies, due to recall bias. However, several publications have demonstrated that using VAS to measure self-recorded outcome following immunotherapy parallels the more objective, and more regularly monitored, symptom and medication score (SMS), up to five years following treatment [21–23]. In addition, with the small sample size of 35 patients used in this study, it is impossible determine whether ILIT is an effective treatment for the general allergic population. However, previous studies with smaller [9] and larger [6] sample sizes show similar effects, which suggests that ILIT has a generalised efficacy. Further, as this study only spanned over one year, long-term efficacy of ILIT is unknown. Nevertheless, previous studies have demonstrated that efficacy of ILIT against grass-pollen-induced rhinoconjunctivitis persists for at least 3 years following treatment [6].

## Conclusions

This double-blind placebo-controlled trial adds to the existing literature and provides further evidence that ILIT is a safe and effective treatment for pollen-induced allergic rhinitis. ILIT circumvents the drawbacks typically associated with SCIT, namely the frequent injections, high risk for adverse events and long duration of treatment, and may therefore represent a novel lower-cost form of AIT. However, larger and longer-term studies, investigating efficacy as well as immunological mechanisms are required to optimise the ILIT treatment regimes. Nonetheless, the present study lends further support for ILIT being a future safe and effective form of AIT. In addition, the data presented suggests that NPT and high affinity allergen-specific IgG_4_ may be valuable predictors for the efficacy of ILIT. If so, this represents a simple way to evaluate the long-term persistence of symptom-reducing effects of ILIT.

## Additional files

Additional file 1:
**Supplementary Methods.** Additional methods not provided in the main text. (DOCX 20 kb) Additional file 2:
**Supplementary Data.** Two tables depicting change in medication use in active and placebo ILIT groups, as well as improved and non-improved sub-groups. (DOCX 16 kb)
