# Supplementary material for: Intralymphatic immunotherapy of pollen-induced rhinoconjunctivitis: a double-blind placebo-controlled trial
Source: Respir Res. 2016 Jan 27;17:10. doi: 10.1186/s12931-016-0324-9 (PMC4728811; doi:10.1186/s12931-016-0324-9)
Supplement: Additional file 1: — Supplementary Methods. Additional methods not provided in the main text. (DOCX 20 kb) [file 12931_2016_324_MOESM1_ESM.docx]

**Intralymphatic immunotherapy of pollen-induced rhinoconjunctivitis: a double-blind placebo-controlled trial**

**Supplementary Methods**

**Methods used for assessment of eligibility**

*Skin prick tests*

SPTs were performed with a standard panel of 11 common airborne allergens (ALK-Abéllo, Horsholm, Denmark) including pollen (birch, timothy, mugwort and ragweed), house dust mite (*Dermatophagoides pteronyssimus* and *D. farinae*), moulds (*Cladosporium* and *Alternaria*) and animal allergens (cat, dog and horse). SPTs were administered on the volar side of forearms, with saline buffer as a negative control and histamine chloride (10 mg/ml) as a positive control. All patients presented a wheal reaction with a diameter of more than 3 mm toward birch and/or grass.

*Blood sampling*

Venous blood was obtained from all 35 participants at baseline (before injection of the allergen), four weeks after the last allergen injection and at the end of the consecutive pollen season. At all time points, blood was collected in tubes containing EDTA (Vacuette® 454209, Greiner Bio-One, Stockholm, Sweden) and levels of allergen-specific IgE and IgG_4_ were determined using the Phadia CAP system (Uppsala, Sweden). A sample containing 4 ml blood was collected was collected in a tube containing EDTA (Vacuette® 454209) and used for flow cytometry analysis.

*Nasal provocations and symptom score*

Before treatment, 4 weeks after treatment and after the end of the consecutive pollen season, study subjects were intranasally challenged with 10,000 SQ-U birch pollen or grass pollen extract per nostril, as described previously [1]. The occurrence and severity of nasal allergic symptoms (itching, secretion and congestion) were recorded by the patients and also scored as positive by the trial staff during the first 30 minutes after challenge. A scale ranging from 0 to 3 (0: no symptoms; 1: mild symptoms; 2: moderate symptoms; 3: severe symptoms) was used. A total symptom score was calculated by adding by combining the scores for itching, secretion and congestion.

**Methods used for assessment of secondary and additional trial outcomes**

*Flow cytometry analyses*

Flow cytometry analyses were performed on a Coulter Epics Xl flow cytometer (Beckman Coulter, Marseille, France). 100,000 – 200,000 events were collected depending on the occurrence of the investigated marker and data was analysed using CxP Analysis software (Beckman Coulter). To ensure flow cytometric standardisation, the voltage settings were updated daily using FlowSet calibration beads (Beckman Coulter). All antibodies were titrated before use, and staining intensity was controlled on a weekly basis.

50µl whole blood was incubated with antibodies against extracellular markers for 20 min at RT. Intracellular staining was performed using the Intraprep^TM^ Permeabilisation Reagent Kit (Beckman Coulter), according to the manufacturer’s instructions. Briefly, cells were fixed and permeabilised using formaldehyde and saponin, respectively. Cells were thereafter stained with antibodies against intracellular antigens for 20 min at RT. Erythrocytes were lysed by mixing with 600µl 0.1 (w/v) formic acid for 3-4 s and the ionic strength was rendered isosmotic by addition of 280 µl 50mM Na_2_CO_3_, 0.20M Na_2_SO_4_ and 0.22M NaCl. Finally, cells were washined in PBS and resuspended in 1% formaldehyde (in isoflow) prior to analysis. Regulatory T-cells were gated as CD4^+^CD25^+^FoxP3^+^.

The following antibodies were used: CD4-PE-Cy5 (13B8.2, Beckman Coulter), CD25-ECD (B1.49.9, Beckman Coulter) and FoxP3-FITC (PCH101, eBioscience, San Diego, CA, USA). The following isotype controls were used: msIgG-FITH (eBiosicence), msIgG1-PE (P3, eBioscience) and msIgG2b-ECD (MPC-11, Beckman Coulter).

*IgG_4_ Affinity ELISA*

Ninety six-well, flat-bottomed polystyrene plates were coated with 5000 SQ-U/ml grass pollen allergen at 4°C overnight. Following a 30-minute block with 2% bovine serum albumin (Sigma-Aldrich), patient serum samples diluted 1:2 1:8 or 1:32 were added and incubated overnight at room temperature. Varying concentrations (0.6, 1.0, 2.0M) of ammonium thiocyanate (NH_4_SCN, Sigma-Aldrich) were subsequently added and incubated for 20 minutes at RT. Allergen-IgG_4_ complexes were detected with an alkaline phosphatase-linked mouse monoclonal anti-human IgG_4_ antibody (Abcam, Cambrdige, UK). The reaction was visualised with alkaline phosphatase yellow (pNPP) liquid substrate (Sigma-Aldrich) and quantitatively measured at 405nm.

1. Ekman AK, Fransson M, Rydberg C, Adner M, Cardell LO: **Nasal challenge with LPS stimulates the release of macrophage inflammatory protein 1alpha.** *Int Arch Allergy Immunol* 2009, **149:**154-160.
