# Supplementary material for: Intralymphatic immunotherapy of pollen-induced rhinoconjunctivitis: a double-blind placebo-controlled trial
Source: Respir Res. 2016 Jan 27;17:10. doi: 10.1186/s12931-016-0324-9 (PMC4728811; doi:10.1186/s12931-016-0324-9)
Supplement: Additional file 2: — Supplementary Data. Two tables depicting change in medication use in active and placebo ILIT groups, as well as improved and non-improved sub-groups. (DOCX 16 kb) [file 12931_2016_324_MOESM2_ESM.docx]

**Intralymphatic immunotherapy of pollen-induced rhinoconjunctivitis: a double-blind placebo-controlled trial**

**Supplementary Data**

**Additional Table 1**: Change in allergy medication use in patients receiving active ILIT or placebo ILIT

|  | Active ILIT | | | Placebo ILIT | | |
| --- | --- | --- | --- | --- | --- | --- |
|  | Decreased | Increased | No Change | Decreased | Increased | No Change |
| Anti-Histamines | 29% | 12% | 59% | 46% | 0% | 54% |
| Corticosteroid Nasal Spray | 25% | 13% | 62% | 22% | 22% | 56% |
| Eye Drops | 33% | 17% | 50% | 22% | 11% | 67% |

**Additional Table 2**: Change in allergy medication use in improved or non-improved sub-groups of patients receiving active ILIT

|  | Improved | | | Non-Improved | | |
| --- | --- | --- | --- | --- | --- | --- |
|  | Decreased | Increased | No Change | Decreased | Increased | No Change |
| Anti-Histamines | 38% | 0% | 62% | 14% | 29% | 57% |
| Corticosteroid Nasal Spray | 67% | 0% | 33% | 14% | 29% | 57% |
| Eye Drops | 38% | 12% | 50% | 25% | 13% | 62% |
